# Comparing Psychometric Characteristics of a Computerized Cognitive Test (BrainCheck‐Assess) against the Montreal Cognitive Assessment (MoCA)

**DOI:** 10.1002/alz.091834

**Published:** 2025-01-03

**Authors:** Duong Huynh, Reza Hosseini Ghomi, Bin Huang, Mary Patterson

**Affiliations:** ^1^ BrainCheck, Inc, Austin, TX USA; ^2^ Frontier Psychiatry, Billings, MT USA; ^3^ BrainCheck, Austin, TX USA

## Abstract

**Background:**

Previous validation studies demonstrated that BrainCheck Assess (BC‐Assess), a computerized cognitive test battery, can reliably and sensitively distinguish individuals with different levels of cognitive impairment (i.e., normal cognition (NC), mild cognitive impairment (MCI), and dementia). Compared with other traditional paper‐based cognitive screening instruments commonly used in clinical practice, the MoCA is generally accepted to be among the most comprehensive and robust screening tools, with high sensitivity/specificity in distinguishing MCI from NC and dementia. In this study, we examined: (1) the linear relationship between BC‐Assess and MoCA and their equivalent cut‐off scores, and (2) the extent to which they agree on their impressions of an individual’s cognitive status.

**Method:**

A subset of participants (N = 55; age range 54‐94, mean/SD = 80/9.5) from two previous studies who took both the MoCA and BC‐Assess were included in this analysis. Linear regression was used to calculate equivalent cut‐off scores for BC‐Assess based on those originally recommended for the MoCA to differentiate MCI from NC (cut‐off = 26), and dementia from MCI (cut‐off = 19). Impression agreement between the two instruments were measured through overall agreement (OA), positive percent agreement (PPA), and negative percent agreement (NPA).

**Result:**

A high Pearson correlation coefficient of 0.77 (CI = 0.63 ‐ 0.86) was observed between the two scores. According to this relationship, MoCA cutoffs of 26 and 19 correspond to BC‐Assess scores of 89.6 and 68.5, respectively. These scores are highly consistent with the currently recommended BC‐Assess cutoffs (i.e. 85 and 70). The two instruments also show a high degree of agreement in their impressions based on their recommended cut‐offs: (i) OA = 70.9%, PPA = 70.4%, NPA = 71.4% for differentiating dementia from MCI/NC; (ii) OA = 83.6%, PPA = 84.1%, NPA = 81.8% for differentiating dementia/MCI from NC.

**Conclusion:**

This study provides further validation of BC‐Assess in a sample of older adults by showing its high correlation and agreement in impression with the widely used MoCA.

